# Subwavelength Grating Waveguide Structures Proposed on the Low-Cost Silica–Titania Platform for Optical Filtering and Refractive Index Sensing Applications

**DOI:** 10.3390/ijms23126614

**Published:** 2022-06-14

**Authors:** Muhammad A. Butt, Cuma Tyszkiewicz, Katarzyna Wojtasik, Paweł Karasiński, Andrzej Kaźmierczak, Ryszard Piramidowicz

**Affiliations:** 1 Institute of Microelectronics and Optoelectronics, Warsaw University of Technology, Koszykowa 75, 00-662 Warszawa, Poland; andrzej.kazmierczak@pw.edu.pl (A.K.); ryszard.piramidowicz@pw.edu.pl (R.P.); 2 Department of Optoelectronics, Silesian University of Technology, Ul. B. Krzywoustego 2, 44-110 Gliwice, Poland; cuma.tyszkiewicz@polsl.pl (C.T.); katarzyna.wojtasik@polsl.pl (K.W.); pawel.karasinski@polsl.pl (P.K.)

**Keywords:** sol–gel dip coating, SWG waveguide, refractive index sensor, low-cost platform

## Abstract

The sol–gel dip-coating method is a cost-efficient way for the realization of thin films on a planar substrate. In this work, high-quality, low-loss, and low-surface roughness silica–titania thin films are deposited on a glass substrate with the sol–gel dip-coating method. This platform works in the visible to near-IR wavelength ranges and can be useful for several eye-catching photonic components. The paper is comprised of two parts: the first part deals with the development of a low-cost silica–titania waveguide system, whereas the second part provides detail on the numerical modeling of the SWG waveguide filter and SWG waveguide FP-sensor design. The SWG waveguide NIR-stopband filter can achieve an ER of >40 dB and 3-dB bandwidth of 110 nm designed at optimized parameters. The SWG waveguide-FP structure proposed in this work act as a refractive index sensor where the sensitivity is ~120 nm/RIU by reducing the width of the waveguide. This sensitivity can be further enhanced by reducing the waveguide height. We believe that this work is quite important for the realization of low-cost integrated photonic devices based on the silica–titania platform developed via the sol–gel dip-coating method.

## 1. Introduction

Due to their potential optical utilization, materials such as silica (SiO_2_), titania (TiO_2_), and silica–titania (SiO_2_-TiO_2_) developed via the sol–gel method have been widely studied [1]. They have been exploited to make planar, rib, and ridge waveguides for optical connection with good optical features and low optical transmission losses [2,3,4]. The sol–gel method is straightforward and low-cost [5]. It can be deposited in large areas and does not need to be processed at high temperatures. It permits the creation of thin films with outstanding thermal and mechanical stability as well as great optical performance. An appropriate refractive index contrast between the substrate and the guiding layer is required for a working waveguide. With the proper adjustment of the set-off precursors and solvents, in addition to the molar ratio of the compounds and the thermal treatment temperature, it is possible to fine-tune the refractive index and thickness of the films produced by the sol–gel process. These waveguides may be doped with rare-earth elements, laser dyes, and other organic compounds, allowing SiO_2_ and TiO_2_ materials made by the sol–gel process to be employed in optical amplifiers, laser-active media, and sensing applications [6]. Several research groups have demonstrated optical waveguides based on the SiO_2_-TiO_2_ platform for a variety of eye-catching applications in recent years [7,8,9,10].

Subwavelength gratings (SWGs) have been a critical component in the fabrication of viable, high-performance integrated silicon photonics devices, for instance, low-loss fiber-to-chip couplers [11], selective filters [12], modulators, sensors [13,14], and ultra-broadband waveguide couplers [15]. The cross-section of an SWG waveguide is equivalent to that of a ridge waveguide, but it features periodic refractive index modulation along the propagation direction of light. Specific wavelength bands can be directed as a Bloch waveguide mode due to the periodicity of the subwavelength grating. SWG waveguide with a certain grating period or duty cycle (hereafter represented as *DC*) may be well-organized for each given wavelength band to be transmitted. The SWG waveguides are appealing because they allow the modification of the guided mode’s effective index and dispersion properties [16]. SWG waveguides are composed of periodic silicon segments that are smaller than the propagation wavelength, removing diffraction effects and acting like a typical waveguide made up of complementary birefringent material [17]. The lithographic manipulation of the material refractive index and dispersion is possible thanks to the etched structural geometry.

EM-wave propagation in periodic media is defined by the Bloch–Floquet principle [18]. A mode’s propagation may be divided into three wavelength areas when designed for a certain grating period (*ᴧ*): (i) The wavelength regime (λ_Bragg_) when the Bloch wave is leaky, and the waveguide scatters a portion of the mode power. (ii) In the wavelength range comparable to the photonic bandgap (PBG) when the frequency bands are below the diffraction zone, Bragg reflections occur. Furthermore, the propagation light cannot be phase-matched to the radiation modes effectively. Instead of a sequential grating structure, reflections constructively add together, producing powerful Bragg reflection [19]. As a result, the incoming light is gradually attenuated and reflected to the input port. (iii) The sub-wavelength regime, in which the wavelength-to-period ratio is equal to (λ/Λ) > 2n_eff_. It is identical to the spectral area above λ_Bragg_ where the waveguide operation is the same as a typical waveguide. As the effects of diffraction and reflection are minimal, the mode is lossless at Λ << λ. It is comparable to how electrons are distributed in periodic potentials in semiconductor materials. SWG waveguides are gaining a lot of interest because the geometric characteristics of the SWG waveguide core may be changed to modify the mode propagation [20,21].

In this work, we have developed a low-cost and flexible technique based on the sol–gel dip-coating method for the deposition of high-quality and low-loss SiO_2_-TiO_2_ thin films. This optical system is highly attractive as it works from visible to near-infrared (NIR) ranges and can be employed in several interesting applications. As a proof of concept, a broadband NIR-filter and FP-sensor established on the SWG waveguide are numerically simulated which offers encouraging results. With the development of nanoimprint lithography in near future, integrated photonic devices based on this platform will be straightforwardly mass-produced at a high-resolution, low-cost, and single fabrication step. Table 1 compares the properties of the three main waveguide platforms to the SiO_2_-TiO_2_ thin-film technology, demonstrating their neck-and-neck competitiveness. When this technology matures, we believe the SiO_2_-TiO_2_ platform will be able to outperform the present costly and difficult-to-manage waveguide technologies in the long term [1].

## 2. Advancement of Low-Cost SiO_2_-TiO_2_ Platform

**Figure 1:**
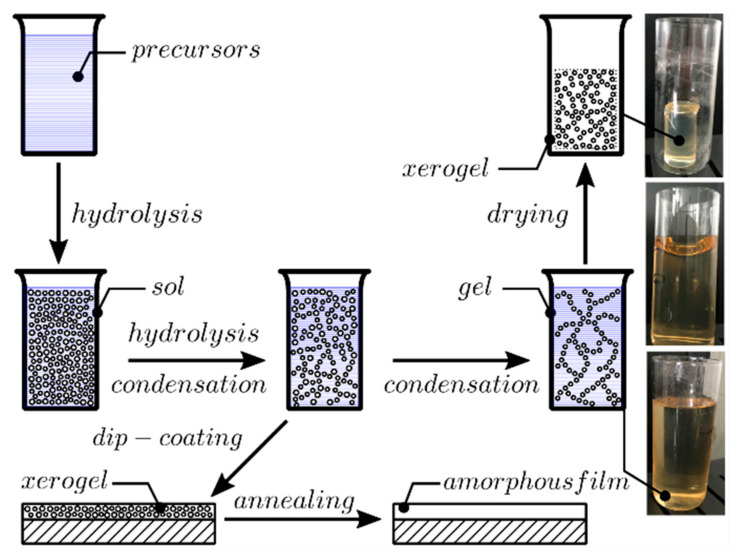
Schematic view of the fabrication process of SiO_2_-TiO_2_ waveguide films on BK7 glass substrates utilizing the sol–gel method and dip-coating technique.

The sol–gel method in combination with the dip-coating technique allows the fabrication of waveguide films in single or multiple coating regimes. This section covers the fabrication process and results of the characterization of single and double-coated SiO_2_-TiO_2_ waveguides on BK7 glass substrates. This process is schematically presented in Figure 1.

Tetraethoxysilane (TEOS) and titanium (IV) ethoxide (TET) were used as the precursors to SiO_2_ and TiO_2_, respectively. These two reagents were purchased from Sigma-Aldrich. The other reagents used in sol synthesis were deionized water, anhydrous ethanol (EtOH), and hydrochloric acid (HCl). The latter was used as the catalyst for concurrently running reactions of hydrolysis and condensation. The process of preparing the SiO_2_-TiO_2_ sol was carried out in two stages. In the first stage, the hydrolysis of each precursor was carried out separately. Then, the solutions were mixed in the appropriate proportion and the process was continued. A sol fabricated this way was aged in tightly closed vessels at the temperature of 180 °C. The films were deposited on BK7 glasses slides with the dimensions of 76 × 26 × 1 mm^3^.

**Figure 2:**
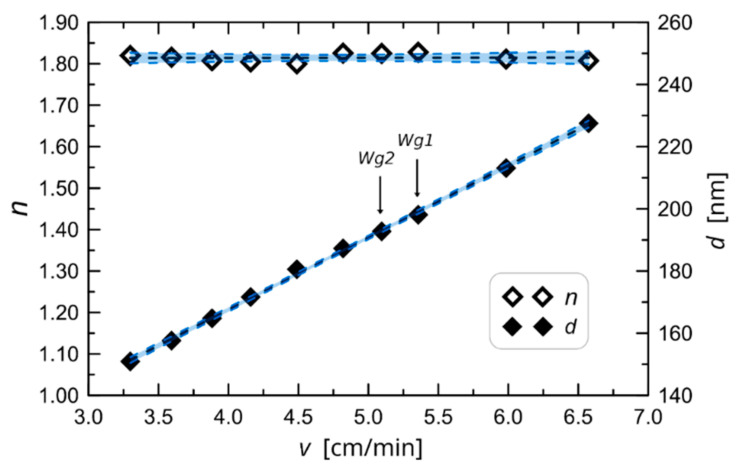
The experimentally determined characteristic of waveguide film thickness *d* and refractive index *n* against change in substrate withdrawal speed from the sol *v*.

To determine the fundamental technological characteristic that shows the relationship between both thickness *d* and refractive index *n* of waveguide films, concerning substrate withdrawal speed from sol *v*, the number of films were deposited on glass substrates, each at a different speed *v*. After deposition, the films were annealed at the temperature of 500 °C for 60 min. Finally, the thickness and refractive index of those films were measured using the monochromatic, multiangle ellipsometer Sentech SE 400 adv operating at the wavelength of 632.8 nm. The resulting characteristics are presented in Figure 2.

Both characteristics: *d*(*v*) and *n*(*v*) are very well approximated by linear functions: *d*(*v*) = *A_d_ v* + *B_d_* and *n*(*v*) = *A_n_ v* + *B_n_*. The coefficient of these functions is presented in Table 2. Linear regression confidence bands for statistical significance level *α_s_* = 0.05 are shown in Figure 1.

One can see from the characteristics presented in Figure 2 that in the presented range of *v*, a maximum thickness that can be obtained in a single coating process is about 230 nm. Fabrication of thicker films is possible with the multiple coating process, which is significant in that after each coating the sample must be annealed. For this paper, we have fabricated the set of ten SiO_2_-TiO_2_ waveguides in the single coating process, which has enabled the determination of the characteristics presented in Figure 2, and a few extra films in the double coating process. Apart from the monochromatic ellipsometry, waveguide films were characterized using transmission and reflection spectroscopy, atomic force microscopy (AFM), and optical transmission losses were measured using the streak method. In the paper, the results are presented for two waveguides that are marked with symbols: Wg1 and Wg2. The first one was fabricated in a single coating process for the withdrawal speed of *v* = 5.4 cm/min. The second waveguide, fabricated in the double coating process, was withdrawn at speed of *v* = 5.1 cm/min. The main reason for selecting only two waveguides was dictated by a willingness to compare single and double-coated waveguides for which the length of streaks of the scattered light, from which optical transmission losses were determined, are comparable and if possible. The refractive index and thickness values of the waveguide Wg2 measured using the SE400 ellipsometer and calculated from linear equations whose coefficients are given in Table 2 are presented in Table 3.

**Figure 3:**
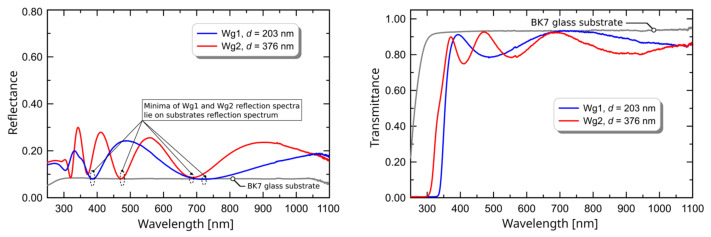
Measured transmission and reflectance spectra of waveguides Wg1 and Wg2.

Measured values of refractive index and thickness belong to confidence intervals presented in Figure 2. Considering the application of planar waveguides for the design of optical sensors which use the evanescent wave spectroscopy technique [22], it is worth remembering that step-index waveguides with high refractive index contrast are the best selection for this kind of application. That is because they have maximum homogeneous or surface sensitivities [23,24]. Analysis of transmission of reflectance spectra allows relatively easy to conclude whether a dielectric film is homogeneous or not. However, the film must have a thickness in a range allowing for observation of interference minima and maxima. As it was shown in [4], when maxima of transmission spectra and minima of reflectance spectra that are far from an absorption edge lay on a transmission and reflectance spectrum of the substrate, then the film is optically homogeneous—it has a uniform (step-index) distribution of refractive index in a direction perpendicular to its surface. The transmission and reflectance spectrum of Wg1 and Wg2 are presented in Figure 3. One can observe that the maxima of transmission spectra for wavelengths greater than 450 nm lie on a spectrum of the BK7 glass substrate, as well as the minima of reflectance spectra for wavelengths greater than 400 nm. This confirms that considering the distribution of the refractive index in the direction perpendicular to the surface, investigated SiO_2_-TiO_2_ films are homogeneous.

**Figure 4:**
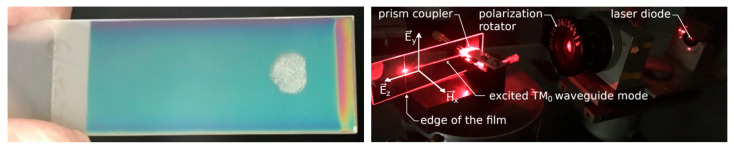
The picture on the **left** presents the waveguide film illuminated with white light revealing its interference color. The picture on the **right** presents the streak of scattered light accompanying propagation of the TM_0_ mode.

The uniformity of waveguide film thickness on scales comparable to dimensions of the substrate is important for application development. The left-hand-side picture in Figure 4 presents the waveguide film illuminated with white light. Its color, resulting from interference, is uniform, excluding areas near the edges of the sample. It bespeaks for uniformity of the waveguide film thickness. The right-hand side picture in Figure 4 presents silica–titania waveguide film fabricated on the BK7 glass substrate in which a TM_0_ mode, excited by using a prism coupler, is propagating. The experiment was conducted more than 10 years after the fabrication of that waveguide. As a result, we can witness the chemical stability of the manufactured thin films. One can observe that the streak is uniform down to the edge of the waveguide film.

The surface morphology of the silica–titania waveguide films was analyzed using the AFM method. From the central part of both investigated waveguides, rectangular samples of width 27 mm and length 20 mm were cut. The picture of the Wg2 in the AFM holder is presented in Figure 4 (left) and the picture of the Wg2 in the prism coupler is shown in Figure 4 (right).

**Figure 5:**
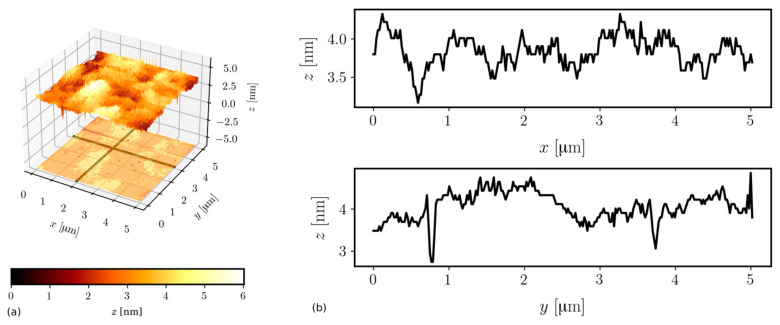
An AFM image (**a**) and roughness profiles (**b**) of the Wg1 waveguide.

**Figure 6:**
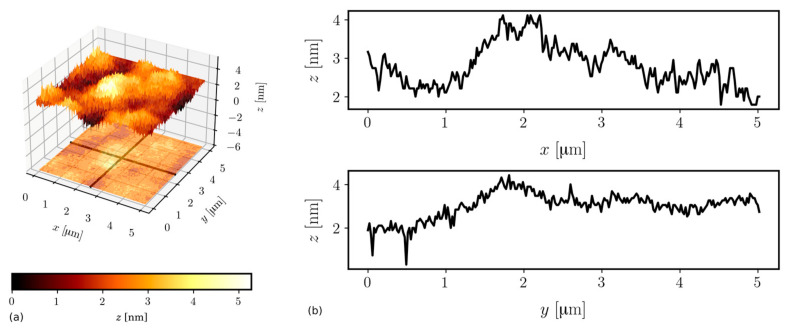
An AFM image (**a**) and roughness profiles (**b**) of the Wg2 waveguide.

Measurements were carried out using a system N_TEGRA Spectra (NT-MDT). The HA_NC (NT-MDT) silicon cantilever operated in semi-contact mode at the frequency of 220.07 kHz. The nominal curvature radius of a tip is 10 nm. Images were obtained from scans having areas of size 5 × 5 μm^2^. For each sample, three scans were performed at different spots. The NOVA 1.0.26.1644 (NT-MTD) software was applied to correct the tilt of the sample and calculate the average surface roughness σ. The surface topology of Wg1 and Wg2 are shown in Figure 5 and Figure 6, respectively.

Fabrication of waveguide films having as small as possible surface roughness is particularly desired if those films are to have low transmission optical losses. That is because the scattering of a light wave guided in a waveguide is the main source of optical losses if material absorption is negligible. That last condition is fulfilled for silica–titania SiO_2_-TiO_2_. According to the model elaborated by Lacey and Payen [25], optical losses are linearly increasing with the squared surface roughness σ on both waveguide film interfaces to a substrate and a cover. The roughness of the substrate is generally higher and depends on the quality of glass substrates. The roughness of the interface to cover depends mainly on the quality of the sol. The roughness of SiO_2_-TiO_2_ films is very low. The average values of σ and their maximum absolute deviations determined from three points on the Wg1 and Wg2 are σ_Wg1_ = 0.58 ± 0.29 [nm] and σ_Wg2_ = 0.63 ± 0.33 [nm]. One can see that the average surface roughness of the film fabricated in a double coating process is only slightly higher. To validate whether the surface roughness is increasing because of repetition of the coating process, or the result reported here is a mere accident, requires further systematic study.

**Figure 7:**
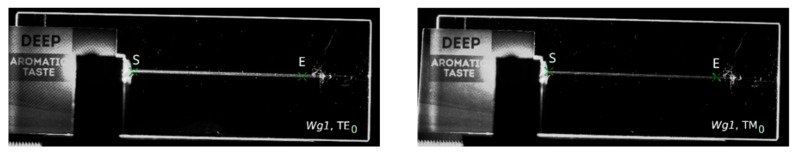
Streaks of scattered light accompanying propagation of TE_0_ and TM_0_ modes in the Wg1 waveguide. The endpoints (S and E) of scan lines are marked by a green cross.

**Figure 8:**
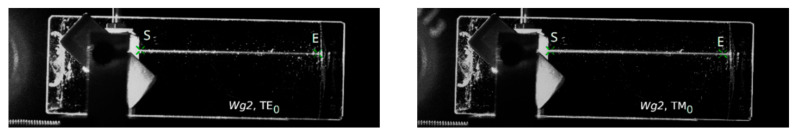
Streaks of scattered light accompanying propagation of TE_0_ and TM_0_ modes in the Wg2 waveguide. The endpoints (S and E) of scan lines are marked by a green cross.

**Figure 9:**
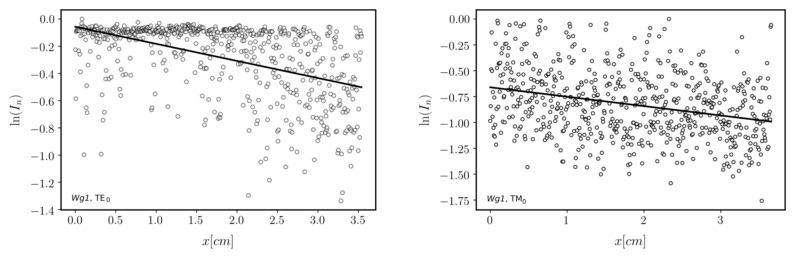
The linearized distribution of normalized light intensity for linear scans selected from light streaks is presented in Figure 7 (Wg1).

**Figure 10:**
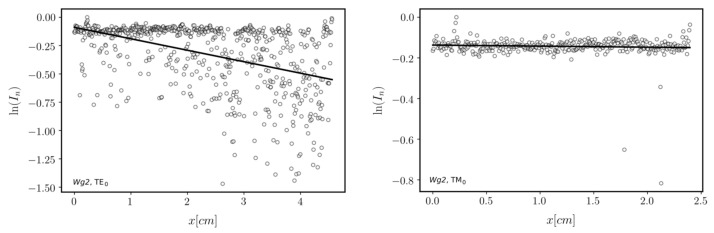
The linearized distribution of normalized light intensity for linear scans selected from light streaks is presented in Figure 8 (Wg2).

Finally, fabricated waveguides were verified by coupling light into them using a prism coupler and observing streaks of scattered light accompanying the propagation of fundamental waveguide modes: TE_0_ and TM_0_. The picture of the Wg_2_ in the prism coupler holder is presented on the right in Figure 4. A laser diode operating at the wavelength *λ* = 677 nm was used as a light source. Optical transmission losses of guided modes can be determined assuming that the distribution of light intensity in such a streak is directly proportional to the distribution of light intensity of the guided mode. Assuming that the light intensity is exponentially decaying $I\left(x\right)=I\left(0\right)\cdot \exp\left(-\mu x\right)$, the attenuation coefficient, which is equal to α=4.343·μ, can be calculated by fitting a linear function to the distribution of the natural logarithm of normalized light intensity *I_n_*. The latter was obtained for each linear scan selected from captured images of light streaks by dividing intensity values by the maximum value in the given streak. The images are shown in Figure 7 and Figure 8. The lines’ endpoints (S and E) are marked by a green cross. The linearized distributions of normalized light intensity are presented in Figure 9 and Figure 10. Streaks of scattered light were registered using a scientific grade, monochromatic camera Thorlabs 1501 equipped with a lens MVL35M23—35 mm EFL. The camera produces grayscale pictures with a resolution of 1392 × 1040 pixels and 14-bit depth. The light intensity *I* expressed in arbitrary units changes from 0 to 16,383.

Determined values of optical transmission losses α and their deviations uα for both investigated waveguides and both polarizations are presented in Table 4. One can observe that optical losses for the Wg2 waveguide are smaller. That is because this waveguide is thicker than Wg1. Fundamental modes are better confined to the Wg2. The results show the density of optical power on the interface to the cover is smaller and in consequence, optical transmission losses are smaller. This experiment proves that fabricated SiO_2_-TiO_2_ waveguide films, both single-coated and double-coated, have very good transmission properties.

## 3. Device Design and Numerical Model

**Figure 11:**
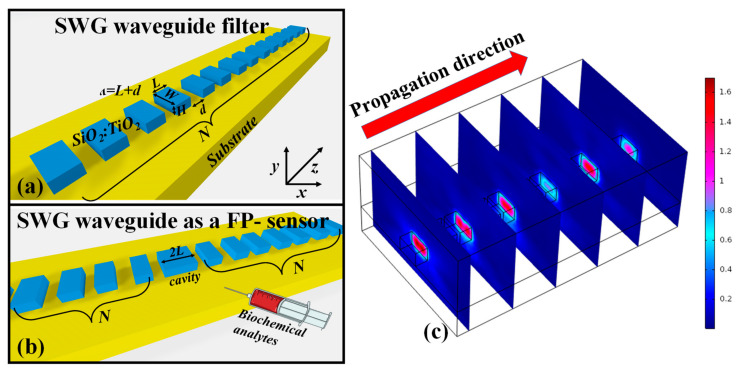
Schematic representation of (**a**) SWG waveguide for NIR-filtering application, (**b**) SWG waveguide as an FP-sensor, (**c**) E-field distribution in the SWG waveguide for the operational wavelength of 810 nm taken at different points along the propagation direction. The geometric parameters used in the analysis are as follows: *L* = 400 nm, *DC* = 80%, *H* = 400 nm, *W* = 800 nm.

In this section, the potential filtering and sensing applications based on the SiO_2_-TiO_2_ platform have been numerically demonstrated. Figure 11a,b depict the SWG waveguide NIR-filter and SWG waveguide FP-sensor architecture, respectively. SWG waveguides have enticed a significant amount of interest as they allow for customized EM-wave propagation by adjusting period (*ᴧ*), which is the sum of *L* and *d*, where *L* is the length of the waveguide segment and d is the distance between two segments. The total length of the waveguide is considered as ((*ᴧ* × *N*) − *d*), where *N* is the total number of periods as shown in Figure 11. The height and width of the waveguide segments are represented as *H* and *W*, respectively. The refractive index of the silica substrate is taken at 1.45 for the operational wavelength of 980 nm. Whereas, the refractive index of the SiO_2_-TiO_2_ thin film is selected from the real part of the effective refractive index, which is dependent on the dimension of the waveguide core. The E-field distribution in the SWG waveguide at different points along the propagation direction is shown in Figure 11c.

The SWG waveguides were modeled using a 2D finite element method (FEM) based on COMSOL Multiphysics 5.5. As a physics interface, the EM-wave frequency domain was utilized. The FEM reduced large elements into smaller components identified as finite elements. This may have been accomplished by performing a space discretization in the space dimensions, which was carried out by generating an object mesh. The detailed meshing was determined by the accuracy of the solution and the computer’s processing power. For the whole waveguide model, we utilized a mesh size of λ/30. Based on our system’s processing speed, this meshing produced accurate simulation results. A domain with an open computation domain was used for wave propagation systems because it allows the EM-wave to flow without back reflections. The open geometry was allotted by using the scattering boundary condition (SBC) at the simulation windows outside borders. At the waveguide’s input port, an *x*-oriented plane wave was excited. Furthermore, the “parametric sweep” function is utilized to simulate various geometric aspects of the waveguide concerning a certain wavelength range.

**Figure 12:**
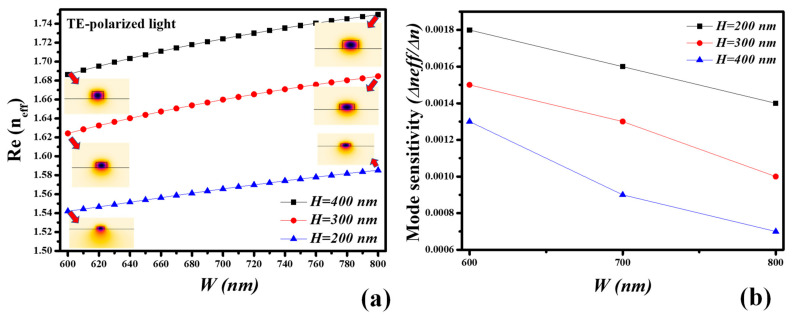
Modal characteristics, (**a**) real part of n_eff_ of SiO_2_-TiO_2_ ridge waveguide for TE-polarized light at operation wavelength of 980 nm, (**b**) mode sensitivity analysis.

The real part of the effective refractive index (n_eff_) of the transverse electric (TE) mode is intended for the operation wavelength of 980 nm by varying the dimensions of the ridge waveguide. This examination is imperative to find the waveguide geometry for the best sensing performance. The height (*H*) of the SiO_2_-TiO_2_ waveguide core is considered as 200 nm, 300 nm, and 400 nm which is practically realizable via single and multiple dip-coating methods. The width (*W*) of the waveguide core is varied between 600 nm and 800 nm. From Figure 12a, it can be seen that the Re (n_eff_) surges as the *W* and *H* of the waveguide core increase which indicates the better mode confinement in the core. Consequently, the contact of the evanescent field with the ambient medium is less which results in low sensitivity. Therefore, we must choose the waveguide core dimensions near the cut-off wavelength where the maximum evanescent field is present. In Figure 12b, the mode sensitivity analysis is executed by changing the ambient refractive index (ARI) from 1.33 to 1.34, resulting in the shift in the effective index of the TE-mode. The sensitivity is calculated as ∆*n_eff_*/∆*n*. The mode sensitivity of the waveguide core with smaller dimensions is higher than the sensitivity offered by bigger waveguide cores. This indicates the maximum sensitivity of 0.0018 ∆*n_eff_*/∆*n* is attained at the waveguide core of *H* = 200 nm and *W* = 600 nm.

## 4. NIR Filtering Application

The optical band-rejection filters are implemented using a variety of methods. A few of these are described here, for example: In [26], an optical band-rejection filter was established on a multistage μ-ring add-drop filter with an extinction ratio (*ER*) > 50 dB, and bandwidth (BW) of 32 GHz has been proposed. An experimental demonstration of a wide optical band-rejection filter with a modest footprint established on two parallel rows of an anti-symmetric one-dimensional Photonic crystal with a defect in a multimode waveguide is presented in [27]. It has an *ER* of 25 dB and a stopband of 200 nm. Another method for constructing an optical band-rejection filter is to use a cladding modulated anti-symmetric long-period grating in a two-mode silicon waveguide. It has a BW of 4.3 nm and an *ER* of more than 15 dB by adopting a 1040 μm long cladding modulated long-period grating [28]. There are other efforts to develop broadband adjustable optical band-rejection filters established on a multimode 1D Photonic crystal waveguide with an 84 nm BW. The device has a 40 × 1 m^2^ footprint [29]. The device performance of the SWG waveguide NIR filter is discussed in terms of *ER* and BW. *ER* is calculated as

$$ER=10\times \log\left(\frac{P_{out}}{P_{in}}\right),$$

where *P_out_* and *P_in_* are the output and input power, respectively. Additionally, the 3 dB BW is obtained from the transmission spectrum where the power level of the signal declines by 3 dB from its maximum value.

**Figure 13:**
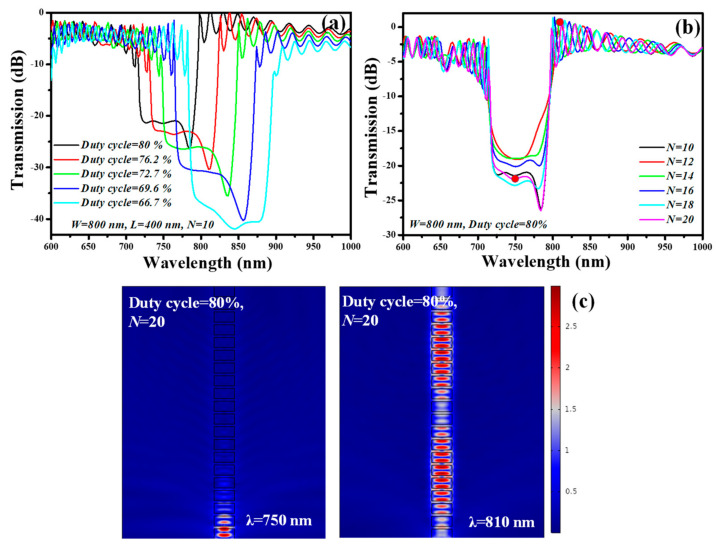
Spectral features of SWG waveguide filter, (**a**) transmission versus *DC*, (**b**) transmission versus *N*, (**c**) E-field distribution at λ= 750 nm occurring in the stopband (**left**) and *λ* = 810 nm (**right**) occurring above λ_Bragg_.

The transmission spectrum of the SWG waveguide NIR-filter versus the *DC* is plotted in Figure 13a. The geometric parameters such as *W*, *H*, *L*, and *N* are fixed at 800 nm, 400 nm, 400 nm, and 10, respectively, as the device is designed for filtering applications. Therefore, the choice of *W* = 800 nm and *H* = 400 nm is based on our previous analysis where we determined the low mode sensitivity of the structure. Whereas, the *DC* varies between 66.7% and 80%, resulting in the enhancement in the *ER* and the 3-dB BW of the filter. Thus, it is determined that the stopband region can be adjusted by choosing the proper *DC*. Furthermore, the influence of *N* on the transmission spectrum is analyzed in Figure 13b. *N* plays a momentous role in augmenting the *ER* of the stopband region without altering the BW and the region of the stopband. The summary of the *ER* and 3 dB BW dependent on the *DC* and *N* is presented in Table 5. The E-field distribution of the SWG waveguide filter in the stopband (indicated by red dots in Figure 13b) and above the λ_Bragg_ (indicated by red dots in Figure 13b) is presented in Figure 13c (left) and Figure 13c (right), respectively.

*DC* plays an imperative role in enhancing the *ER* and 3dB-BW of the SWG waveguide stopband filter. From Table 5, it can be seen that, as the *DC* varies from 80% to 66.7%, the *ER* increases from ~19 dB to ~41.9 dB. *ER* can also be modified by adjusting the *N* at a constant *DC*. The 3 dB BW of the filter upsurges from 79 nm to 110 nm as the *DC* changes from 80% to 66.7%.

## 5. Sensing Application

**Figure 14:**
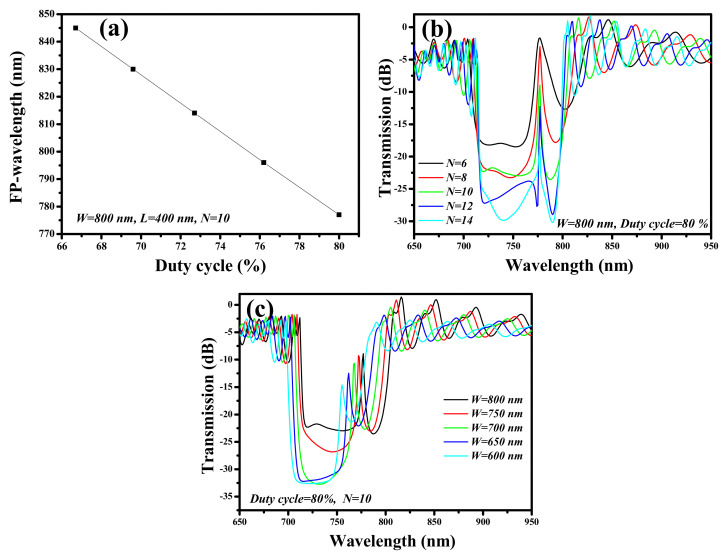
Spectral characteristics of the SWG waveguide-FP structure, (**a**) FP-wavelength versus *DC*, (**b**) transmission spectrum versus *N*, (**c**) transmission spectrum versus *W*.

A cavity of size 2*L* is inserted in the center of the SWG waveguide to create an FP-sensor structure as shown in Figure 11b. The geometry of the SWG waveguide-FP structure needs to be optimized for the best sensing performance. The *DC*, *N*, and *W* are important parameters that should be carefully handled while designing such devices. At first, the FP-wavelength occurring in the stopband region is determined concerning the *DC* of the device structure which is varied between 66.7% and 80%, while keeping *W*, *H*, *L*, and *N* constant at 800 nm, 400 nm, 400 nm, and 10, respectively. The surrounding medium is air (*n* = 1.0). As shown in Figure 14a, the FP-wavelength shows a blueshift as the *DC* increases from 66.7% to 80%, which signifies that the desired spectral properties can be obtained by varying the *DC* of the device.

In the second step, the influence of *N* on the spectral properties of the SWG waveguide-FP structure is determined as shown in Figure 14b. The remaining geometric parameters such as *W*, *H*, and *DC* are fixed at 800 nm, 400 nm, and 80%, respectively. It can be seen that, as *N* increases from 6 to 14, the *ER* of the stopband significantly increases from ~17 dB to ~30 dB with a reduction in the full width at half maximum (FWHM) of the FP-wavelength. Therefore, a precise selection of *N* is important, however, a bigger *N* can lead to a bigger device footprint.

For sensing applications, a large overlap of an evanescent field with the ambient medium is required for enhanced sensing devices. Therefore, *W* plays an important role in increasing the sensitivity and if the device is designed close to the cut-off dimensions of the waveguide, then maximum sensitivity can be obtained. The transmission spectrum of the SWG waveguide-FP structure is plotted for *W* varying in the range of 600 nm and 800 nm, as shown in Figure 14c. The remaining geometric parameters such as *H*, *DC*, and *N* are fixed at 400 nm, 80%, and 10, respectively. The FP-wavelength performs a blueshift as *W* decreases from 800 nm to 600 nm. From Figure 14a,c, we can see that *DC* and *W* have direct control over tuning the FP-wavelength.

**Figure 15:**
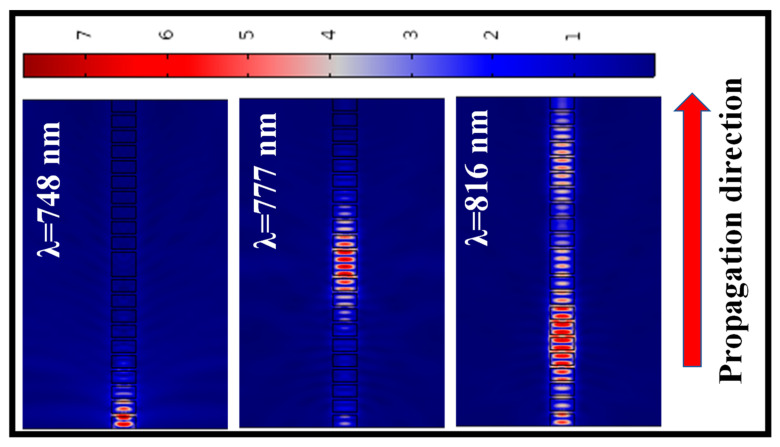
E-field distribution in the FP-structure at *λ* = 748 nm (**left**), *λ* = 777 nm (**middle**), and *λ* = 816 nm (**right**). Geometric parameters used in the analysis are as follows: *DC* = 80%, *W* = 800 nm, *N* = 10.

The E-field distribution in the SWG waveguide NIR-filter structure at Bragg wavelength (λ = 748 nm), FP wavelength (λ = 777 nm), and above Bragg wavelength (λ = 816 nm) is shown in Figure 15 (left), Figure 15 (middle), and Figure 15 (right), respectively. This indicates the behavior of EM wave in the stopband, FP-cavity, and the passband.

**Figure 16:**
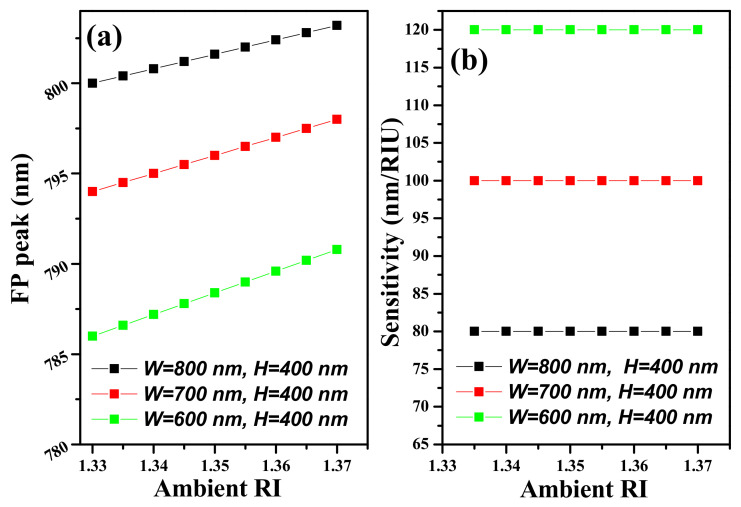
Spectral characteristics of SWG waveguide-FP structure, (**a**) FP peak versus ARI, (**b**) Sensitivity.

To demonstrate the sensing performance of the FP-structure, the wavelength interrogation method has been used. Sensitivity (*S*) is the ratio between the change in the FP-wavelength and the change in the ARI, which is expressed as:

*S* = ∆*λ*/∆*n*,

where ∆*λ* and ∆*n* are the change in FP-wavelength and the change in ARI, respectively. The ambient index varies between 1.33 and 1.37 with a step size of 0.005. The FP-wavelength and sensitivity of the device having different dimensions concerning the changing ARI are shown in Figure 16a,b, respectively. The *S* of the FP-structure varies between 80 nm/RIU and 120 nm/RIU at *H* = 400 nm by reducing the *W* from 800 nm to 600 nm. Additionally, this sensitivity can be further enhanced to 20–30% by reducing *H* from 400 nm to 200 nm. The sensitivity characteristics of the SWG waveguide FP-structure are summarized in Table 6. In [30], Fard et al. used 90 nm SOI strip waveguide height to enhance bulk sensitivity.

## 6. Anticipated Fabrication Methods

The waveguide system based on the sol–gel dip-coating method is cost-efficient and shows great potential to be utilized in several interesting applications. Integrated photonic devices based on such platforms can be inexpensive if the low-cost fabrication method can also be utilized. Currently, we are working and mastering the development of the conventional fabrication method which includes photolithography (for patterning) and reactive ion etching (RIE) of the patterned structures [31]. Additionally, we aim to develop direct nanoimprinting for the development of low-cost integrated photonic devices [32]. This approach entails printing patterns in a liquid sol–gel with a flexible polymer mold, then separating the pattern and transferring it to the substrate. Pattern preparation using photolithography is the initial stage in the process, which precisely involves the deposition of a photoresist on a silicon substrate and exposure of the sample via a lithography mask. The design is then soaked with a liquid polymer (PDMS). The stamp is now ready to pattern transfer by impressing the structures on the previously produced sol–gel layer when the PDMS curing is complete. Silicon master mold provided the flexible mold, which can be composed of PDMS. The pattern of the PDMS layer is brought into contact with a substrate and sol–gel during nanoimprinting. Capillary molding is then used to fill the PDMS mold’s patterns with liquid sol–gel. UV irradiation was used to harden the sol–gel formations at first. The PDMS is then removed, leaving solid microstructures on the substrate’s surface. The final hardening of the nanostructures is conducted at 500 °C for 1 h to eliminate organics and water after the initial hardening.

## 7. Conclusions

In this work, a sol–gel dip-coating method is utilized for the realization of high-quality SiO_2_-TiO_2_ thin films on a glass substrate. The coating method is highly cost-effective and provides thin films with low surface roughness and low losses, which is ideal for the waveguiding purpose. Due to the ongoing experimentations on the fabrication process which includes reactive ion etching and nanoimprinting method, numerical modeling of highly attractive SWG waveguides for filtering and sensing applications have been presented via the finite element method. The SWG waveguide structure is modeled for a NIR-stopband filter which offers an *ER* > 40 dB and 3-dB BW of 110 nm with a total device length in the range of 5.9 μm and 11.9 μm. By modifying the SWG waveguide geometry, an FP structure has been designed to be employed in refractive index sensing applications. The sensitivity of the device is in the range of 80 nm/RIU to 120 nm/RIU depending on the width of the waveguide. This sensitivity can be further enhanced by reducing the height of the SWG waveguide. The optical system proposed in this paper has the potential to be employed in several noteworthy applications due to its extraordinary optical, physical, and chemical properties.

## Figures and Tables

**Table 1:** Comparison of optical, physical, and chemical properties of InP, SOI, SiN, and SiO_2_-TiO_2_ platforms.

	InP	SOI	SiN	SiO_2_-TiO_2_
Refractive index	3.4	3.42	2.0	1.6–2.2
Spectral range [μm]	NIR	1–1.65	Visible to NIR	Visible to NIR
Propagation loss (dB/cm)	>0.4	<0.1	<0.1	~0.1
Realization of thin films	LP MOCVD	Wafer bonding	LPCVD	Sol–gel
Implementation costs	High	High	High	Low
Technological maturity	High	High	High	Under development
Cost efficiency	Moderate	Very high	Moderate	Very high
Refractive index modification	No	No	Yes (only for SiO_x_N_x_)	Yes (1.1–2.2)
Chemical resistance	Low	Low	Moderate	Very high

**Table 2:** Coefficients of linear functions approximating experimental relationships *d*(*v*) and *n*(*v*) and their standard deviations.

*A_d_* 10^−7^ [min.]	*B_d_* [nm]	*A_n_* [RIU·min.·cm^−1^]	*B_n_* [RIU]
23.170339	74.930564	0.000231207	1.8132074
*u*(*A_d_*)	*u*(*B_d_*)	*u*(*A_n_*)	*u*(*B_n_*)
1.1792197	0.24416098	0.016142592	0.0033423722

**Table 3:** Comparison of the refractive index and thickness values for the waveguide Wg2 which were measured and theoretically predicted from the characteristics *d*(*v*) and *n*(*v*).

	Measured	Calculated
*d* [nm]	381.4	386.2
*n*	1.7943	1.8144

**Table 4:** Measured values of optical transmission losses in Wg1 and Wg2 waveguides.

	Wg1		Wg2	
	TE_0_	TM_0_	TE_0_	TM_0_
*α* [dB/cm]	0.54	0.39	0.44	0.07
*u_α_* [dB/cm]	0.04	0.05	0.03	0.01

**Table 5:** Measured values of optical transmission losses in Wg1 and Wg2 waveguides.

*DC* (%)	Total Length of Device (Microns)	*ER* (dB)	3-dB BW (nm)	*N*
80	~4.9–9.9	~19–26.35	~79	10–20
76.2	~5.15–10.4	~30.34–30.5	~89	10–20
72.7	~5.4–10.9	~33.9–35.5	~99	10–20
69.6	~5.65–11.4	~36–40.3	~105	10–20
66.7	~5.9–11.9	~37.3–41.9	~110	10–20

**Table 6:** Sensing performance of the SWG waveguide-FP structure versus *W*.

*L* (nm)	*d* (nm)	*W* (nm)	*S* (nm/RIU)
400	100	800	80
400	100	700	100
400	100	600	120

## Data Availability

Not applicable.

## Abbreviations

Silica–titania	SiO_2_-TiO_2_
Subwavelength grating	SWG
Extinction ratio	*ER*
Full width at half maximum	FWHM
Reactive ion etching	RIE
Atomic force microscopy	AFM
Polydimethylsiloxane	PDMS
Bandwidth	BW
Duty cycle	*DC*
Effective refractive index	n_eff_
